# Lazy Sundays: role of day of the week and reactivity on objectively measured physical activity in older people

**DOI:** 10.1186/s11556-019-0226-1

**Published:** 2019-10-27

**Authors:** Jochen Klenk, Raphael Simon Peter, Kilian Rapp, Dhayana Dallmeier, Dietrich Rothenbacher, Michael Denkinger, Gisela Büchele, the ActiFE Study Group, T. Becker, B. Böhm, K. Scharffetter-Kochanek, J. Stingl, W. Koenig, M. Riepe, R. Peter, H. Geiger, A. Ludolph, C. von Arnim, G. Nagel, G. Weinmayr, J. M. Steinacker, R. Laszlo

**Affiliations:** 1 Institute of Epidemiology and Medical Biometry, Ulm University, Helmholtzstr 22, 89081 Ulm, Germany; 2 Department of Clinical Gerontology, Robert-Bosch-Hospital, Auerbachstr 110, 70376 Stuttgart, Germany; 3 IB University of Applied Sciences Berlin, Study Center Stuttgart, Paulinenstraße 45, 70178 Stuttgart, Germany; 4 Bethesda Geriatric Clinic, Zollernring 26, 89073 Ulm, Germany

**Keywords:** Physical activity, Older people, Research methods, Measurement, Reactivity

## Abstract

**Background:**

The aim of this study was to assess the effect of day of the week and wearing a device (reactivity) on objectively measured physical activity (PA) in older people.

**Methods:**

Walking duration as a measure for PA was recorded from 1333 German community-dwelling older people (≥65 years, 43.8% women) over 5 days using accelerometers (activPAL). Least-square means of PA with 95%-confidence intervals (95%-CI) from multi-level analysis were calculated for each day of the week and each measurement day (days after sensor attachment).

**Results:**

Walking duration on Sundays was significantly lower compared to working days (Sunday vs. Monday-Friday: − 12.8 min (95%-CI: − 14.7; − 10.9)). No statistically significant difference compared to working days was present for Saturdays. The linear slope for measurement day and walking duration was marginal and not statistically significant.

**Conclusions:**

Studies using PA sensors in older people should assess Sundays and working days to adequately determine the activity level of the participants.

## Introduction

The beneficial effects of a physically active life style on various health outcomes have been investigated in many studies [1]. Low levels of physical activity (PA) are negatively associated with several health-related parameters and phenotypes, such as cardiovascular disease, cancer, diabetes, obesity, bone mineral density, and self-perceived well-being [2–4]. During the last decade, body-worn sensors, including accelerometers, have provided objective measurements of real-life PA [5]. However, several methodological aspects have to be considered to precisely assess the amount of PA and to interpret the results correctly [6, 7]. Besides others, day of the week and measurement day (number of days after sensor attachment) may affect results of PA measurements. It has been observed that people are more active at the beginning of a measurement period due to the fact that their activity is recorded [8–11]. This phenomenon is called reactivity to the device. Furthermore, physical activity might not be distributed equally across the week. Several studies reported less activity on the weekend compared to working days [11–13].

Especially in older people, reactivity to the device and the effect of day of the week on PA have not been investigated in large cohorts, yet. Therefore, the aim of the study was to analyse the association between day of the week and measurement day with daily walking duration in a cohort of older people aged 65 years and older.

## Methods

### Study population

The ActiFE-Ulm (Activity and Function in the Elderly in Ulm) study is a population-based cohort study in older people ≥65 years, randomly selected in Ulm and adjacent regions in Southern Germany. Exclusion criteria were: being in residential care, severe deficits in cognition or serious German language difficulties. Between March 2009 and April 2010, 1506 eligible individuals agreed to participate and underwent baseline assessments. The study has been previously described in detail [14]. All participants provided written informed consent, the Ethics Committee of the University of Ulm had approved the study (application no. 318/08 and 50/12) and the study have conformed to the principles embodied in the Declaration of Helsinki.

### Physical activity measurement

PA at baseline was measured using a validated uni-axial accelerometer (activPAL, PAL Technologies Ltd., Glasgow, UK) [15]. The sensor was sealed against water and attached to the thigh using an adhesive tape. Participants were instructed to wear the sensor over 24 h for seven consecutive days. Only days with activity measurements over the full 24 h were considered as a valid day and included in the analysis. Accordingly, the first and the last day of each assessment period were excluded as well as all participants without a valid measurement on a Sunday.

The sensor data was downloaded and processed using a proprietary software from the device manufacturer. Based on the acceleration data, the provided algorithm detects upright posture as well as walking patterns and classified the time-stamped activities into three categories: (1) lying or sitting, (2) standing and (3) walking. The duration of walking at each day served as outcome measure for PA in this study.

### Statistical analysis

Daily walking duration was calculated for each participant and for each available day in minutes (min) per day. Day of the week and measurement day served as independent variables. When discussing day of the week, all days except Saturdays and Sundays were defined as ‘working days’. In all analyses, PA was estimated using multi-level analyses with the subjects on the second-level and measured days on the first-level. For each category, the least-square means with 95%-confidence intervals (95%-CI) of daily walking duration were estimated. All analyses were adjusted for sex, age and weather conditions (daily maximum temperature) as well as for day of the week or measurement day if it was not the main independent variable of the analysis [16]. Calculations were performed using SAS 9.4 and R 3.4.0.

## Results

The study population consisted of 1333 participants (43.8% women) at baseline with a mean age of 75.5 years (SD = 6.5) (Table 1). Overall 7511 complete days of PA measurements were analysed. More than 90% of the participants had at least 5 measurement days.

**Table 1:** Characteristics of study population

	Baseline (*n* = 1333)
Female, n (%)	584 (43.8)
Age (years), mean (SD)	75.5 (6.52)
BMI (kg/m^2^), mean (SD)	27.6 (4.14)
Self-reported comorbidity, n (%)	
Cardiovascular disease	333 (25.0)
Cancer	239 (17.9)
Diabetes	185 (13.9)
Total days measured, n	7511
Persons with ≥5 days measured, n (%)	1216 (91.2)
Average daily walking duration (min), mean (SD)	
Monday	104.2 (48.3)
Tuesday	105.8 (49.5)
Wednesday	108.3 (49.0)
Thursday	108.1 (49.9)
Friday	105.2 (47.8)
Saturday	104.4 (49.8)
Sunday	92.5 (49.5)

*n* number, *SD* standard deviation

**Fig. 1:**
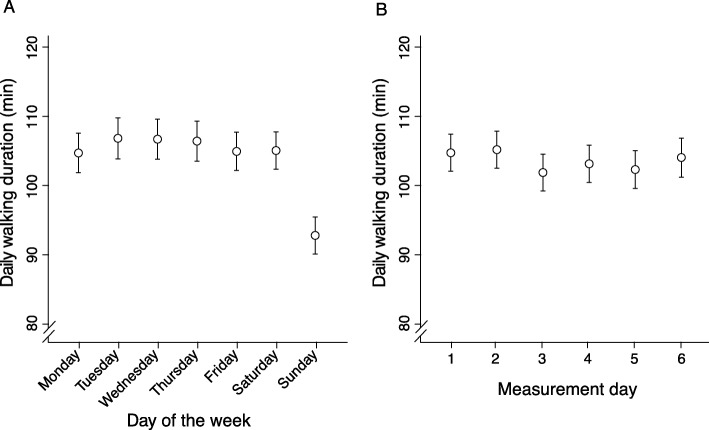
Association between day of the week (**a**) and measurement day (number of days since sensor attachment) (**b**) with daily walking duration adjusted for sex, age, and daily maximum temperature as well as for day of the week (**b**) or measurement day (**a**)

Figure 1a shows the relationship between day of the week and daily walking duration. Daily walking duration was significantly lower on Sundays compared to all other days of the week. The mean difference between Sundays and working days (Monday-Friday) was − 12.8 min (95%-CI: − 14.7; − 10.9). No statistically significant difference compared to working days was present for Saturdays.

The linear slope for measurement day and walking duration (Fig. 1b) was − 0.32 min per day (95%-CI: − 0.80; 0.14).

## Discussion

In this cohort of older persons, we found a statistically significant decrease of walking duration on Sundays, which was more than 10% lower, compared to working days. A difference of walking duration with measurement day (reactivity) was not observed.

Our results confirmed previous findings in different age cohorts that the amount of PA is less on weekends compared to working days [11–13, 17]. In older people Sunday seems to be the most inactive day, as clearly seen in our study. However, we did not find a statistically significant decrease in PA on Saturdays. In Germany, shopping, visit of public authorities, a hairdresser, a bank, or similar operations are not possible on Sundays. This might decrease out-of-home time and thereby lead to the observed decreased walking duration on Sundays.

Regarding reactivity to PA measurement the previously available data have been collected in children, adolescents and middle aged adults and showed inconsistent results [8, 10, 11, 18, 19]. An experimental study in younger adults found that PA was 19% lower if participants were not informed about the function of the sensor and the aim of the measurement, suggesting reactivity to PA measurement in case of the knowledge of the purpose and aim of being under observation [20]. The findings in our cohort suggest that reactivity is not problematic in older people. However, this might be device-specific as the attachment method and size of the activPAL is relatively unobtrusive compared to other devices [21].

The major strengths of our study are the large population-based cohort of older people and the large number of measurement days over 24 h. The main limitation is that the participants were only measured on seven consecutive days. The duration might be too short to observe significant reactivity.

## Conclusions

Studies using sensor-based measurement of PA in older people should include Sundays and working days in the assessment period to adequately determine the activity level of the participants. Furthermore, reactivity does not seem to be clinically relevant by the used measurement device in this cohort of older people.

## Data Availability

The data that support the findings of this study are available from the ActiFE-Ulm study group but restrictions apply to the availability of these data, which were used under license for the current study, and so are not publicly available. Data are however available from the authors upon reasonable request and with permission from the ActiFE-Ulm study group.
