# Correction to: Lazy Sundays: role of day of the week and reactivity on objectively measured physical activity in older people

**DOI:** 10.1186/s11556-019-0232-3

**Published:** 2019-12-05

**Authors:** Jochen Klenk, Raphael Simon Peter, Kilian Rapp, Dhayana Dallmeier, Dietrich Rothenbacher, Michael Denkinger, Gisela Büchele, the ActiFE Study Group

**Affiliations:** 1 Institute of Epidemiology and Medical Biometry, Ulm University, Helmholtzstr 22, 89081 Ulm, Germany; 2 Department of Clinical Gerontology, Robert-Bosch-Hospital, Auerbachstr 110, 70376 Stuttgart, Germany; 3 IB University of Applied Sciences Berlin, Study Center Stuttgart, Paulinenstraße 45, 70178 Stuttgart, Germany; 4 Bethesda Geriatric Clinic, Zollernring 26, 89073 Ulm, Germany

**Correction to: Eur Rev Aging Phys Act (2019) 16:18**

**https://doi.org/10.1186/s11556-019-0226-1**

Following publication of the original article [1], the authors reported an error in the author list. The study group has been listed with the authors in the pdf version. The original article has been corrected. The correct presentation of the authors is shown below.

Jochen Klenk, Raphael Simon Peter, Kilian Rapp, Dhayana Dallmeier, Dietrich Rothenbacher, Michael Denkinger, Gisela Büchele, the ActiFE Study Group.
